# Aortico-left ventricular tunnel experience on three different ages

**DOI:** 10.4103/0975-3583.74265

**Published:** 2010

**Authors:** Turkay Saritas, Nurdan Erol, Abdullah Erdem, Aliriza Karaci, Ahmet Celebi

**Affiliations:** *Department of Pediatric Cardiology, Dr Siyami Ersek Thoracic and Cardiovascular Surgery Center, Istanbul, Turkey*; 1 *Department of Cardiovascular Surgery, Dr Siyami Ersek Thoracic and Cardiovascular Surgery Center, Istanbul, Turkey*

**Keywords:** Aortic insufficiency, ventricular dilatation, aortico-left ventricular tunnel

## Abstract

Aortico-left ventricular tunnel is extremely rare congenital paravalvar communication between the aorta and the left ventricle. Usually it is treated surgically. In addition to the surgery the tunnel can be closed by percutaneous transcatheter intervention in appropriate patients. We present in this paper 7 months, 10 years, and 1,5 months old three male cases with aortico-left ventricular tunnel that were surgically treated and followed up within 7 years in our clinic.

## INTRODUCTION

Aortico-left ventricular tunnel (ALVT) which was defined firstly by Levy is extremely rare congenital paravalvar communication between the aorta and the left ventricle.[1] Abnormalities in proximal parts of coronary arteries or other abnormalities such as aortic and pulmonary valve abnormalities can accompany the tunnel.[2] We present, in this paper, 7-month-, 10-year-, and 1.5-month-old three male cases with ALVT that were surgically treated and followed up within 7 years in our clinic.

## CASE REPORTS

### Cases 1 and 2

**Figure 1:**
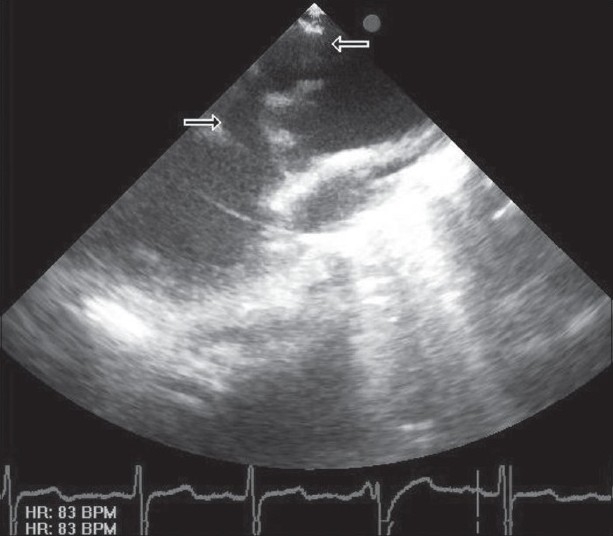
Two-dimensional imaging of the tunnel in transthoracic echocardiographic examination

While systolic and diastolic murmurs, thrill in the mesocardiac area and congestive heart failure findings were observed during physical examination in the 7-month-old case with mild dyspnea, there was no pathological finding other than a fourth-degree systolo-diastolic murmur and trill in the mesocardiac area in the 10-year-old case with complaints of throb, getting tired quickly, and mild dyspnea during effort. Left ventricle dilatation, aortic root dilatation, and the tunnel arising from the upper part of right coronary artery that was connecting aorta to the left ventricle were determined by two-dimensional imaging in a transthoracic echocardiographic examination in both cases [Figure 1]. Systolic and diastolic flow in the tunnel was shown by color Doppler echocardiography as well.

Cardiac catheterization was performed for eliminating coronary abnormality in the 7-month-old case. In this case, ALVT, and also the absence of associated abnormality such as coronary abnormality were shown. The case who was not considered for transcatheter closing put on the anticongestive treatment and referred to the surgery department. In the surgical operation, the orifice of the tunnel extending from the right coronary cusp to the commissure in the left of right coronary artery orifice was seen. This tunnel was opened in a way next to the left coronary cusp–right coronary cusp commissure under the right coronary cusp of aorta.

**Figure 2:**
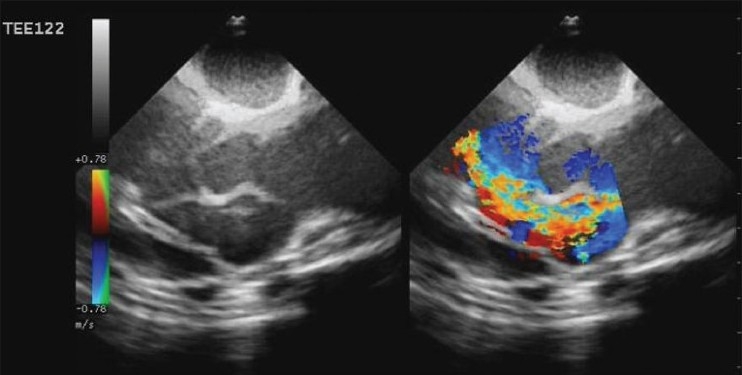
Two-dimensional and color flow imaging of the tunnel in transesophageal echocardiographic examination

In transthoracic echocardiographic examination for the 10-year-old case, it was determined that the narrowest place of the tunnel was 2.8 mm, the widest place of which was 27 mm, and aortic annulus was 21 mm. It was thought that the tunnel could be closed with a suitable device using transesophageal echocardiography by the percutaneous transcatheter method. The morphology of the tunnel was assessed again using transesophageal echocardiography during cardiac catheterization [Figure 2]. In cardiac catheterization and balloon sizing, the tunnel was 31 mm long, the widest place was 29 mm and narrowest place was 14 mm.

It was planned to close the tunnel by a duct occluder with a dimension of 14 × 16 mm^2^. Although the deployment of the device was performed successfully, the device was withdrawn without positioning because of the increase of aortic failure and dropping down easily to the left ventricle. The case was referred to the surgery department.

### Case 3

A 25-week pregnant, 24 years old, woman was referred to our clinic for echocardiographic examination after fetal cardiomegaly was diagnosed by her gynecologist. The fetal echocardiogram (performed at 25 weeks’ gestation) showed a mild cardiomegaly; the left ventricle was slightly hypertrophic, dilated and spheric. The heart rate was 158 bpm, the left ventricular end-diastolic dimension was 17.1 (normal range, 6–11.5) mm, and the right ventricular end-diastolic dimension was 9 (normal range, 6.2–12) mm. No free fluid was found in either the thorax or the abdomen. No obvious insufficiency was detected in the atrioventricular valves; the venous Doppler flow parameters were normal. The global cardiac function was normal. In the apical 5 chamber view, there was detected a considerable insufficiency surrounding the aortic valve from the side of the right ventricle. In the parasternal long-axis view, a tunnel was detected, beginning directly next to the right sinus of Valsalva, extending to the left ventricle and localized on the anterolateral side of the aorta. A color flow exam demonstrated the blood flow inside the tunnel.

The patient was to come in for fetal echocardiography every 2–4 weeks up until labor in addition to routine gynecological exams. During follow-up, no signs of cardiac failure were detected. Due to the mother’s amniotic fluid breaking at 38 week of gestation, a small for gestational age 2300 g male baby was delivered via the cesarean section. The Apgar score at the 1, 5, and 10 minutes was good; upon physical examination no cyanosis or respiratory distress was observed. The precordium appeared hyperdynamic upon inspection. In *cardiac auscultation, a grade* 3/6 holodiastolic murmur was heard over the left precordium. Peripheral pulses were jerky.

**Figure 3:**
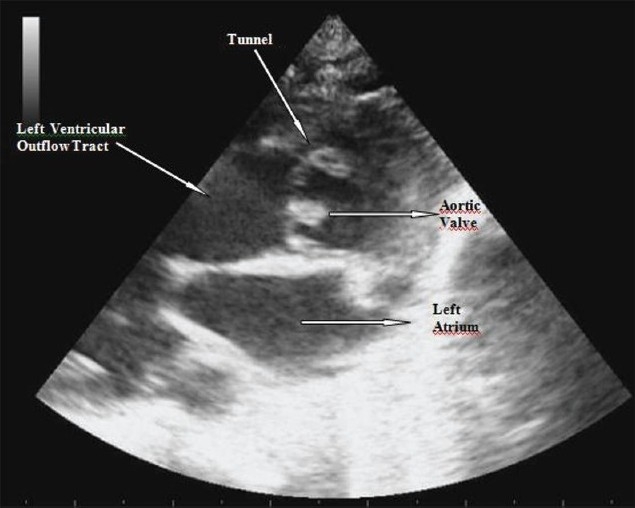
Two-dimensional imaging of the tunnel in transthoracic echocardiographic examination after birth

The patient’s systolic/diastolic gap was wide; he underwent transthoracic 2D and color flow echocardiography which confirmed the diagnosis of ALVT that was suspected during the intrauterine period [Figure 3].

While the aortic valve was tricuspid, no stenosis or deficiency was detected. The patient’s left ventricle was slightly spheric and dilated, its end-diastolic dimension was 22 (normal range, 8.2–17) mm, and the end-systolic dimension was 15 mm. According to an M mode echocardiogram, fractional shortening was 31% and the ejection fraction was 61%. No coronary anomalies were suspected in the patient, and no congenital or dynamic stenosis was found at the right ventricular exit.

The patient was put on digoxin, ACE inhibitors, and diuretics. At the follow-up examination of 1.5-month-old of this case, the left ventricular end diastolic diameter and shortening fraction were measured 33 mm and 29%, respectively, and this patient was operated.

In surgical repair of all cases, firstly, the aortic end was closed with continuing sutures using the Gore-tex patch and prolene sutures and then the ventricular end was closed using the Gore-tex patch and prolene sutures.

In the follow-up of all the three cases, the 7-month-old case, which had the longest follow-up duration 7 years, developed only mild aortic regurgitation. The 10-year-old case was followed for 1 year, and mild aortic insufficiency detected before the surgery persisted without any increment. The third case followed only 3 months, and he has entirely a normal aortic valve. All three patients are still followed up annually by echocardiographic examination for early detection of worsening aortic valve regurgitation.

## DISCUSSION

Aortico–left ventricular tunnel is one of rare aortic regurgitation’s causes, and a large percentage of patients presented with congestive heart failure and almost half of them were asymptomatic.[1] Patients usually refer to hospital with heart failure during the first-year of life. It rarely occurs in the result of the murmur in physical examination.[2] The left ventricular and aortic root dilatation were determined in all cases, there was mild exertional dyspnea in the 10-year-old case but the findings of congestive heart failure were not present. In the 7-month-old case, the findings of congestive heart failure were present.

Aortico-left ventricular tunnel is subdivided into four types by Hovaguimian and his colleagues.[3] In our cases, the aortic orifice of the tunnel arose above or from the right coronary sinus, and they were laying anterolaterally to the ascendent aorta. Fibrous area of the left–right coronary commissure is the place where the tunnel was communicating with the left ventricle. The right coronary aortic leaflet arose from the fibrous tissue; in these cases, the right coronary aortic leaflet cannot be supported due to the fibrous tissue spanning the aortic root.[4] On the other hand, some cases demonstrated that the aortic orifice arose from the left coronary sinus and the tunnel lay posterolaterally to the ascending aorta. In this situation, incision of the tunnel is difficult and the tunnel should be closed through aortic incision.[4] The tunnels in our cases were in conformity with the type 4. The tunnel’s aortic orifice in the 7-month-old case originated from the right coronary cusp and over the right coronary cusp in the 10-year-old and 1.5-month-old cases. Also in all cases, the tunnel’s end was opened to the left ventricle in a way next to the left coronary cusp–right coronary cusp commissure under the right coronary cusp of aorta.

Coronary artery anomalies may be associated with ALVT in 45% of patients. The ostium of the right coronary artery may lie within the tunnel; alternatively, there may be complete absence of the origin of the left or right coronary ostium.[5] There are associated aortic valve anomalies in about 20% of patients ranging from bicuspid aorta to severe dysplasia. Adults may suffer from aortic incompetence or leaflet perforation because of hemodynamic trauma due to the unsupported right coronary cusp, and progressive aortic dilation.[2] Stenosis of the pulmonary valve has been reported around 5%. Compression of the right ventricular outflow tract by the tunnel may produce subpulmonary obstruction. Rarely, both semilunar valves are stenotic.[2] In our cases, associated lesions such as coronary abnormality, aortic or pulmonary valve pathology, or right ventricle outflow obstruction related to the compression of the tunnel were not observed.

The diagnosis is usually made by echocardiography. The cases diagnosed in the intrauterine period were reported. Perinatal diagnose is also employed. In the case of associated cardiac anomalies, cardiac catheterization may be applied for interventions.[5,6] Cardiac catheterization was performed for eliminating coronary abnormality in the 7-month-old case and for closing the tunnel by transcatheter method in the 10-year-old case.

Serious aortic insufficiency leads dilation of the left ventricle and heart failure. Therefore, the lesion that causes aortic regurgitation must be repaired before the left ventricle gets damaged. Fixing aortic regurgitation in children is unique. In adults, aortic regurgitation commonly fixed with the aortic valve replacement and sometimes with the aortic root surgery. As the aortic annulus of the children is smaller than adults, more complex surgery is needed in this group.[7]

Spontaneous closure in only one case with a slit-like tunnel is reported, but patients should be treated even if they have not any symptom. The tunnel is closed by surgery or with a device in appropriate patients. In these patients, ALVT can be closed using a proper device by cardiac catheterization.[2,8] During angiocardiography in the 10-year-old case, we tried to close the tunnel using duct occluder in a dimension of 16/14 mm, but the process was ended without positioning of the device since aortic insufficiency increased and the device’s stabilization was not provided. The aim of the surgery is to close the tunnel. Variable methods are tried for this until today. The surgery method for associated coronary or other abnormalities is varied. It is reported that distortion does not occur in the aortic valve in the technique that both ends of the tunnel are closed by a pericardial patch. A surgery treatment is safe for newborn and infants. Also, the surgeon should decide the technique according to the patient and his own ability.[2,9] Both orifices of the tunnel were closed using the Gore-tex patch in the 7-month-old and 1.5-month-old cases and both ends of the tunnel were closed using primary sutures in the 10-year-old case.

Even if the patients are treated, they should be followed for the tunnel persistence and also aortic aneurysm because of the occurrence of aortic insufficiency in long term and deterioration of the present aortic failure. The incidence of aortic insufficiency in patients with ALVT following surgery ranges from 16 to 60%, and the requirement for aortic valve replacement ranges from 0 to 50%.[9,10] In our 7-month-old case, a mild aortic insufficiency occurred in follow-up after the surgery and the aortic insufficiency persisted in 10-year-old case with initially a mild aortic failure. Aortic insufficiency was not observed in the 1.5-month-old case until now.

## CONCLUSION

In ALVT, coronary artery abnormalities and other associated abnormalities should be observed. In addition to the surgery, the tunnel can be closed by percutaneous transcatheter intervention in appropriate patients. Asymptomatic small tunnel may seldom close spontaneously. After the treatment, all patients should be followed-up for tunnel recurrence, aortic valve incompetence, left ventricular dysfunction, and aortic aneurysm during all life.
